# Focusing on Increasing Velocity during Heavy Resistance Knee Flexion Exercise Boosts Hamstring Muscle Activity in Chronic Stroke Patients

**DOI:** 10.1155/2016/6523724

**Published:** 2016-07-25

**Authors:** Jonas Vinstrup, Joaquin Calatayud, Markus D. Jakobsen, Emil Sundstrup, Lars L. Andersen

**Affiliations:** ^1^National Research Centre for the Working Environment, Lersø Parkalle 105, 2100 Copenhagen, Denmark; ^2^Physical Activity and Human Performance Group, SMI, Department of Health Science and Technology, Aalborg University, 9220 Aalborg, Denmark; ^3^Research Unit in Sports and Health, Department of Physical Education and Sports, University of Valencia, 46010 Valencia, Spain

## Abstract

*Background*. Muscle strength is markedly reduced in stroke patients, which has negative implications for functional capacity and work ability. Different types of feedback during strength training exercises may alter neuromuscular activity and functional gains.* Objective*. To compare levels of muscle activity during conditions of blindfolding and intended high contraction speed with a normal condition of high-intensity knee flexions.* Methods*. Eighteen patients performed unilateral machine knee flexions with a 10-repetition maximum load. Surface electromyography (EMG) was recorded from the quadrics and hamstring muscles and normalized to maximal EMG (nEMG) of the nonparetic limb.* Results*. For the paretic leg, the speed condition showed higher values of muscle activity compared with the normal and blindfolded conditions for both biceps femoris and semitendinosus. Likewise, the speed condition showed higher co-contraction values compared with the normal and blindfolded conditions for the vastus lateralis. No differences were observed between exercise conditions for the nonparetic leg.* Conclusion*. Chronic stroke patients are capable of performing heavy resistance training with intended high speed of contraction. Focusing on speed during the concentric phase elicited higher levels of muscle activity of the hamstrings compared to normal and blindfolded conditions, which may have implications for regaining fast muscle strength in stroke survivors.

## 1. Introduction

Stroke is a leading cause of death and disability among adults worldwide [1]. Despite declines in prevalence, mortality rates, and disability, the global burden of stroke continues to increase due to population growth and aging [2]. Thus, effective interventions to counteract the consequences of stroke are needed. Multifactorial physical therapy is the pillar stone of rehabilitation following stroke. An increasing number of new therapies for rehabilitating the consequences of stroke have emerged during the past decade, for example, virtual reality, mirror therapy, constraint-induced movement therapy, and electromyographic feedback [3, 4]. Historically, high-intensity physical exertion as part of stroke rehabilitation was thought to exacerbate spasticity, meaning that heavy load strength training was often excluded from rehabilitation practices [5]. However, research has firmly established that strength training does not increase spasticity [6–8] and that strength training has the potential to improve function and reduce physical impairment in chronic stroke patients [9–12]. Consequently, the American Heart Association and the American Stroke Association now consider high-intensity strength training as an essential aspect of rehabilitation regimes for stroke patients [13].

Following stroke, bilateral muscle weakness is commonly observed [6, 14]. However, because of the pronounced hemiparesis following stroke, the side of the body contralateral to the lesion will exhibit severe muscle weakness not only compared with healthy individuals but also compared with the ipsilateral body side [7, 9]. In addition, certain lower extremity muscles are more affected and stronger predictors of poststroke mobility than others [9, 15–17]. Knee flexor strength correlates moderately to strongly with walking ability [18] and has been shown to be severely weakened following stroke [9]. Furthermore, decreases in force production with imposed increases in movement velocity are commonly observed in stroke patients [9, 19]. As muscular adaptations are not only contraction- and load- but also velocity-specific [20], it is therefore necessary to include exercise velocities that match those of individual functional requirements. The use of speed/power training and accompanying velocity-specific training adaptations have long been recognized in the strength training literature in healthy subjects and athletes [20–23]. Even when the actual velocity is low but the intended velocity is high, marked improvements of high-velocity muscle strength have been observed [24]. However, the effect of high intended movement speed on muscle activity has not been studied in chronic stroke patients.

Therefore, the aim of this study was to compare levels of muscle activity during conditions of blindfolding and intended high speed contractions with a normal condition of high-intensity knee flexion exercise in chronic stroke patients. We tested the null-hypothesis of no differences in muscle activity between exercise conditions.

## 2. Methods

### 2.1. Subjects

A total of 18 (11 men and 7 women) patients with cerebrovascular injuries in the chronic stage (>6 months after injury) at the Center for Rehabilitation of Brain Injury, Copenhagen, Denmark, participated in two sessions, consisting of (1) familiarization and (2) an experimental protocol. All referrals to the study were made by a physiotherapist at the Center for Rehabilitation of Brain Injury, having screened the patients for eligibility at admission. All participants were medically stable, motivated, taking part in ongoing resistance training at least twice a week, and cleared for strenuous physical exercise. Furthermore, they had been physically active as part of their rehabilitation since the brain injury and more recently (mean 1.4 ± 1.3 years) participating in progressive resistance training at the Center of Brain Injury. The criteria for inclusion were a chronicity of more than 6 months and a moderate-to-severe hemiparesis with unilateral weakness. Exclusion criteria were alcohol or substance abuse, resting blood pressure above 160/100 mmHg, psychiatric diseases, and any progressive diseases.  Table 1 shows the demographics of the participants.

### 2.2. Ethics

All participants were informed about the purpose and content of the study and gave their written informed consent. Before participating in the experimental protocol, the subjects received additional information by email and were verbally informed at the familiarization session. The study conformed to the Declaration of Helsinki and was approved by the Local Ethical Committee (H-3-2010-062).

### 2.3. Experimental Design

#### 2.3.1. Exercise Equipment and Description

The knee flexion exercises were performed unilaterally in the seated knee flexion machine (TechnoGym Isotonic Line with Power Control, TechnoGym SpA, Gambettola, FC, Italy) in 3 different scenarios with the same absolute load: (1) normal, (2) focusing on speed in the concentric phase, and (3) without visual feedback. During the latter scenario the participants were blindfolded. The normal and blindfolded exercises were performed in a controlled manner (each repetition lasting ~3 seconds which was controlled by counting out loud), whereas the participants were instructed to perform the concentric phase as fast as possible in the speed scenario. All exercises were performed in full available range of motion (ROM) for 3 repetitions at a 10 repetition max (RM) load, with the participants being allowed to use grip support. In the same population, we have previously reported muscle activity of the quadriceps and hamstrings during knee extension and flexion utilizing machine equipment compared with elastic resistance [25], lower limb muscle activity during leg press and bodyweight exercises (Vinstrup et al., unpublished observation), and forearm muscle activity during finger flexion and extension exercises (Vinstrup et al., unpublished observation).

#### 2.3.2. Familiarization and Experimental Protocol

At the first session participants were familiarized with the exercises used in the experimental protocol, and the 10 RM load was determined. As the participants were used to performing the seated knee flexion exercise the current 10 RM was located within 3 attempts, with 1-minute rest between sets. The load was accepted when the participant could perform no more than 10 repetitions while maintaining technique. At the second session the EMG apparatus was applied on one side at a time and fixated with adhesive tape (Fixomull, BSN Medical GmbH, Hamburg, Germany), after which the subject was asked to perform various movements to confirm comfort and strength of the application. The order of exercises and legs was randomized and counterbalanced. Each subject chose blindly by picking a piece of paper, hereby receiving an unknown exercise order. No injuries, complaints, or dissatisfaction of any kind was reported during the experimental protocol.

#### 2.3.3. Maximal Voluntary Isometric Contraction (MVC)

At the end of the experimental protocol isometric MVCs were performed in the seated knee flexion machine. Two MVCs were performed for each leg and the highest value was used for later analysis. The participants were instructed to exert maximal force for 5 seconds and were allowed 1 minute of rest between sets. Strong verbal encouragement was given during each trial.

#### 2.3.4. EMG and Inclinometer Signal Sampling and Analysis

Before placing the electrodes (Blue Sensor N-00-S, Ambu A/S, Ballerup, Denmark) the skin was cleaned, shaved, and prepared with scrubbing gel (Acqua gel, Meditec, Parma, Italy) to lower skin impedance [26, 27].

EMG signals were recorded from 4 muscles: biceps femoris (BF), semitendinosus (ST), vastus medialis (VM), and vastus lateralis (VL). A bipolar surface EMG configuration (Neuroline 720 01-K, Medicotest A/S, Ølstykke, Denmark) with an interelectrode distance of 2 cm was used [25, 28]. The EMG electrodes were connected directly to wireless probes that preamplified the signal (gain 400) and transmitted data in real-time to a nearby 16-channel PC-interface receiver (TeleMyo DTS Telemetry, Noraxon, Arizona, USA). The sampling rate was set to 1500 Hz with a bandwidth of 10–500 Hz to avoid aliasing. The resolution of the signals was 16 bits. The common mode rejection ratio was >100 dB.

During later analysis all raw EMG signals obtained during MVCs as well as during the exercises were digitally filtered, consisting of (1) high-pass filtering at 10 Hz and (2) a moving root-mean-square (RMS) filter of 500 ms. For each individual muscle, peak RMS EMG of the 3 repetitions performed was determined, and the average value of these 3 repetitions was normalized to the maximum maximorum of the nonparetic leg [25, 26]. Because stroke patients cannot maximally activate the paretic side, this normalization was performed as the best alternative in order to make muscle activity comparison between limbs possible.

#### 2.3.5. Sample Size Calculation

Sample size calculation was performed prior to the study and showed that 16 subjects in this paired design were sufficient to achieve a statistical power of 80% at a minimal relevant difference of 10%, a Type I error probability of 1%, and assuming a standard deviation of 10% based on previous research in our lab [29].

### 2.4. Statistical Analysis

For each muscle and leg separately, a linear mixed model (Proc Mixed, SAS version 9, SAS Institute, Cary, NC) was used to determine if differences existed between exercises (fixed factor). The mixed procedure inherently handles missing data. Normalized EMG (nEMG) of each respective muscle was included in the model as dependent variable. Subject was entered in the model as a random factor. Values of nEMG are reported as least square means (LSM) and 95% confidence interval (95% CI) unless otherwise stated. Differences between exercises are stated as LSM percentage points of nEMG and 95% CI. *p* values < 0.05 were considered statistically significant.

## 3. Results

Mean 10 RM load during the knee flexion exercise was 22.6 kg (SD 7.8) and 13.8 (SD 6.8) for the nonparetic and paretic leg, respectively (*p* = 0.0019). No adverse events occurred during any of the exercises.

For the paretic leg, the speed condition showed higher values of muscle activity compared with the normal and the blindfolded conditions for both biceps femoris and semitendinosus (*p* < 0.05). Likewise, the speed condition showed higher values of co-contraction compared to the normal and blindfolded conditions for the vastus lateralis (*p* < 0.05). For the vastus medialis, the speed and normal conditions showed higher values of co-contraction compared with the blindfolded condition (*p* < 0.05).

No differences were observed between exercise conditions for the nonparetic leg (Table 2).

## 4. Discussion

This study investigated the effects of performing seated knee flexions blindfolded or with a focus on speed compared with a normal condition of knee flexion on hamstring and quadriceps muscle activity. The main finding was that focusing on high movement speed during the seated knee flexion exercise led to higher muscle activity of the hamstrings compared with normal and blindfolded conditions. This pattern was only observed for the paretic leg. Therefore, our initial null hypothesis is partially rejected.

Our study provides novel insight into the effect of intended movement velocity in chronic stroke patients, effectively contributing to the knowledge base within stroke rehabilitation. We show that increasing movement speed increases agonist muscle activity of the paretic, but not the nonparetic, leg during knee flexions. This is in contrast to healthy individuals where ballistic contractions are known to induce higher levels of muscle activity compared with slow-speed contractions [30]. The reason for this difference between legs can only be speculated upon, but it illustrates that stroke patients will likely benefit from focusing on speed during the concentric phase when exercising the paretic leg. It can be hypothesized that the paretic side is more sensitive to higher velocities due to detraining and/or weakness, whereas the nonparetic side may require even higher velocities or greater loads to increase muscle activation. In addition, antagonist coactivation differed between exercise conditions, consistently showing lower values of muscle activity during the blindfolded condition. This could merely reflect the differences in agonist muscle activity. However, abnormal coactivation patterns [31–33], the need for additional stabilization to ensure joint integrity during high speeds [34], and the potential effect of no visual feedback could also have contributed to these differences [4, 35–37].

The uneven distribution of muscle strength across lower limb muscles and movement speeds could partially explain the awkward gait pattern seen in stroke patients and why the functional use of the paretic leg is often impaired despite adequate isometric and slow concentric muscle strength [38, 39]. A recent intervention study showed that fast concentric (240°/s) knee flexor strength of the paretic leg was nondetectable, that is, zero percentage of the nonparetic leg [9]. In general, that study showed that higher velocity of contraction was associated with more pronounced muscle weakness. This seems to highlight the need for muscle- and velocity-specific rehabilitation.

Previous studies evaluating muscular and functional effects of strength training after stroke have almost exclusively focused on traditional training paradigms, that is, machine based exercises with fixed ranges of motion and controlled movement speeds [40, 41]. This uniform training approach may not be optimal for a number of reasons. First, the need for individually tailored task-specific interventions has been highlighted in the stroke literature [42]. Second, as muscular adaptations are contraction-, muscle-, and velocity-specific [9, 43], some exercise modalities and/or movement speeds are likely to result in a greater functional carryover effect than others. Thus, an unused potential for optimizing stroke rehabilitation paradigms may still exist. Exercise guidelines for healthy individuals and athletes are quite elaborate, emphasizing speed/power training with focus on improving velocity-specific functional movements [44, 45]. In addition, resistance exercise performed with higher movement speeds has been shown to produce more repetitions at given intensities in healthy individuals [46]. In the present study, heavy load strength training with high intended velocity was well tolerated and led to higher levels of muscle activity. Collectively, this suggests that a combination of training diversity and specificity could be included in rehabilitation programs for stroke patients to improve treatment success.

Another reason for incorporating task-specific strength training into stroke rehabilitation is the vital necessity of adequate muscle strength in a broad array of everyday movements. First, a certain minimum level of muscle strength is required to perform several activities of daily living [47]. Second, lower limb muscle strength is often the limiting factor in preventing falls in the elderly population [48]. Third, increasing muscle strength of certain lower limb muscles, including the knee flexors, has been shown to improve walking ability in stroke patients [18, 49]. In this context, the specific movement speeds which are practiced during training could be of great importance: a number of daily activities require actions performed at greater speeds than those practiced during training, and the ability to rapidly produce force has been shown to be essential in preventing falls in the elderly [50, 51].

There are several ways to include specificity and variation in stroke rehabilitation. Elastic resistance exercises are examples of convenient and cost-effective alternatives to the traditional machine exercises found only in rehabilitation facilities and would allow for greater exercise variation, speed, and specificity while maintaining high levels of muscle activity [25, 52]. Furthermore, elastic resistance exercises may allow for a faster start of the rehabilitation period, as this training equipment does not require the patient to commute to designated training facilities. However, interventions evaluating the efficacy of task-specific elastic resistance exercises in relation to walking ability and other functional outcomes are needed. Regardless of the chosen exercise modality, this study illustrates that chronic stoke patients can perform fast contractions safely during isolated exercises and highlights the fact that increasing movement speed poses an easy and attractive way to induce overload and improve rehabilitation practices.

## 5. Conclusion

Chronic stroke patients are capable of performing heavy resistance training with high intended speed of contraction. Focusing on speed during the concentric phase elicited higher levels of muscle activity of the hamstrings compared to normal and blindfolded conditions, which may have implications for regaining fast muscle strength in stroke survivors. Future interventions should investigate the rehabilitative effect of velocity-specific strength training on walking ability and relevant functional outcomes such as work ability.

## Figures and Tables

**Table 1:** Demographics.

Variable	Mean	SD
Age (years)	56.8	(7.6)
Body mass (kg)	71.1	(16.5)
Height (cm)	174.4	(8.9)
Blood pressure, systolic/diastolic (mmHg)	129/81	(11/11)
Sex (male/female)	11/7	

10 RM load (kg) (non/par)	22.6/13.8	(7.8/6.8)
Side of lesion (left/right)	8/10	
Time since injury (years)	3	(3)

**Table 2:** Normalized EMG values for the biceps femoris, semitendinosus (agonists), vastus lateralis, and vastus medialis (antagonists) during knee flexion. Values are presented as least square means (LSM) ± 95% CI and represent % of maximum values. “*∗*” denotes a significant difference compared with the “speed” condition whereas “#” indicates a significant difference between “normal” and “blindfold.”

Muscle	Exercise	Nonparetic leg		Paretic leg	
		Mean (95% CI)	*p* value	Mean (95% CI)	*p* value
Biceps femoris	Speed	60 (49–71)		22 (15–30)	
	Normal	59 (48–71)	NS	19 (12–27)	*∗*
	Blindfold	59 (48–70)	NS	17 (10–25)	*∗*
Semitendinosus	Speed	62 (51–74)		24 (13–35)	
	Normal	61 (50–73)	NS	22 (10–33)	*∗*
	Blindfold	61 (49–73)	NS	21 (10–32)	*∗*

Vastus medialis	Speed	7 (5–9)		7 (5–9)	
	Normal	7 (5–9)	NS	6 (4–8)	#
	Blindfold	8 (5–10)	NS	4 (2–6)	*∗*
Vastus lateralis	Speed	5 (3–7)		8 (5–10)	
	Normal	6 (4–8)	NS	6 (3–8)	*∗*
	Blindfold	6 (4–8)	NS	5 (2–7)	*∗*

Speed: knee flexions performed with a fast concentric phase.

Normal: knee flexions performed in a controlled manner.

Blindfold: knee flexions performed blindfolded.

NS: not significant.
